# Supplementary material for: Insulin Can Delay Neutrophil Extracellular Trap Formation In Vitro—Implication for Diabetic Wound Care?
Source: Biology (Basel). 2023 Aug 3;12(8):1082. doi: 10.3390/biology12081082 (PMC10452400; doi:10.3390/biology12081082)
Supplement: Supplementary file 1 [file biology-12-01082-s001.zip › biology-2505194-supplementary/biology-2505194-supplementary-R1/biology-2505194-supplementary-R1.pdf]

Full WB images

Figure 4 in the main manuscript

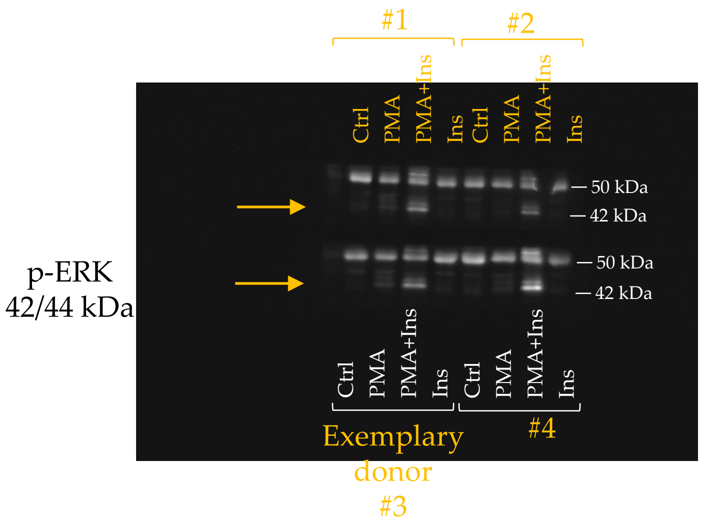

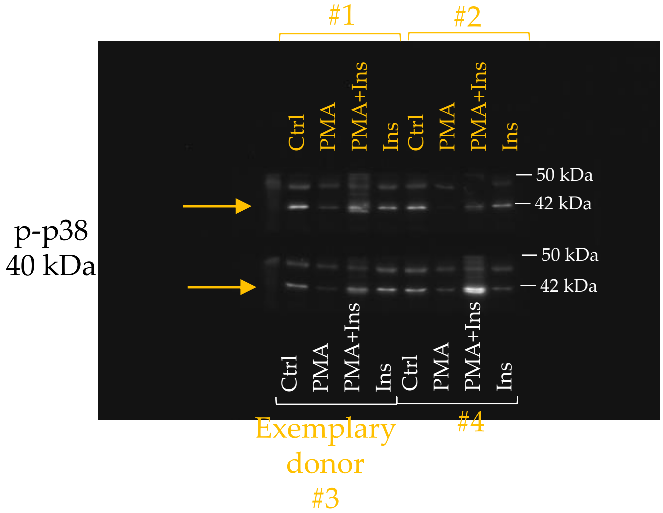

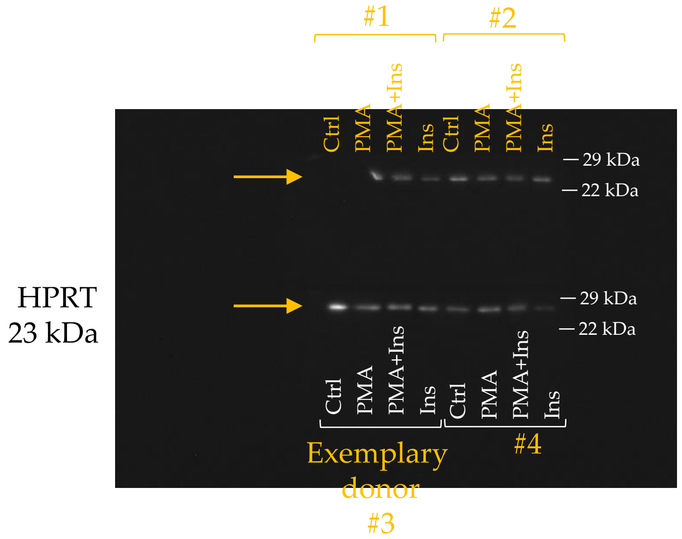

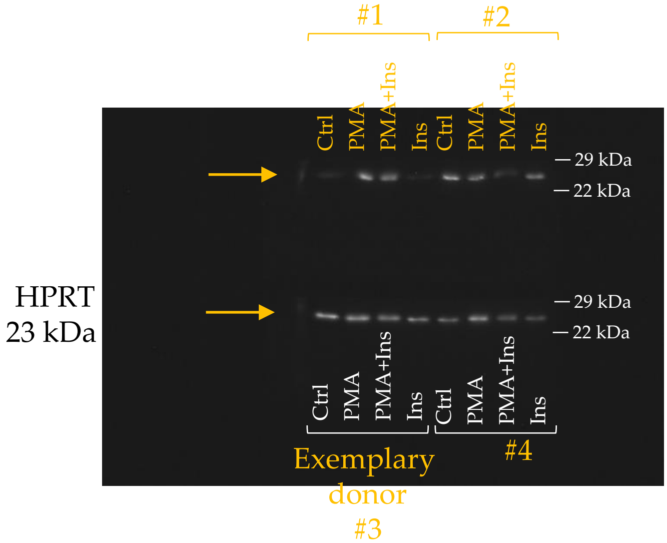

<u>Exemplary donor shown in the main manuscript (Figure 4)</u>

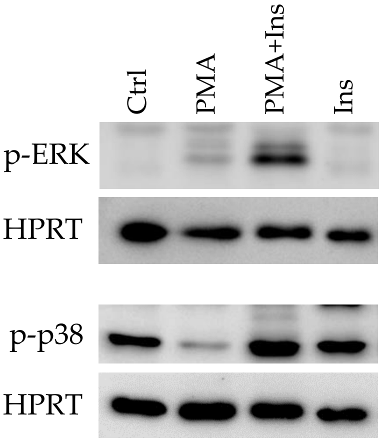

Full Western Blot images for Figure 4E:
Detection of phosphorylated MAPK levels (phospho-p38, left and phospho-ERK, right) by Western Blot after 2 h stimulation of neutrophils.
Yellow arrows indicate right band, size markers are on the right side of the image.
The exemplary donor shown in the manuscript is highlighted and the corresponding figure shown on the left.
PMA: 100 nM, Ins: Insulin 160 IU/L

Figure 4 in the main manuscript

MPO
72 kDa

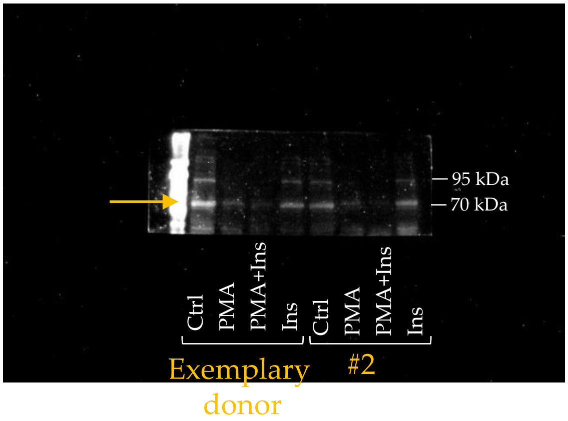

PAD4
67 kDa

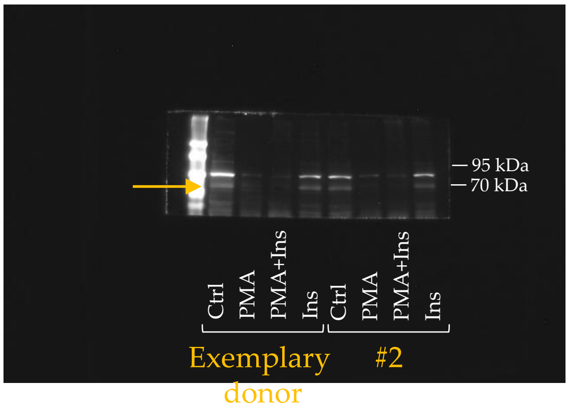

cit-H3
17 kDa

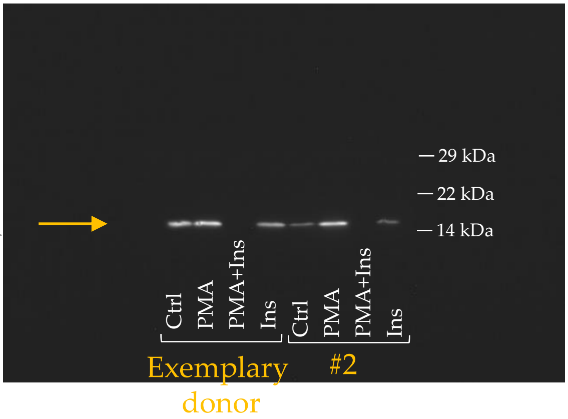

HPRT
23 kDa

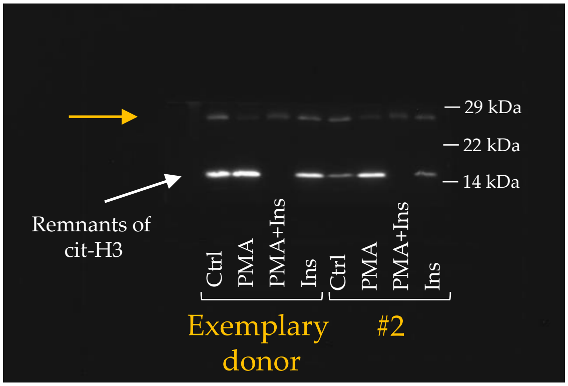

<u>Exemplary donor shown in the manuscript (Figure 4)</u>

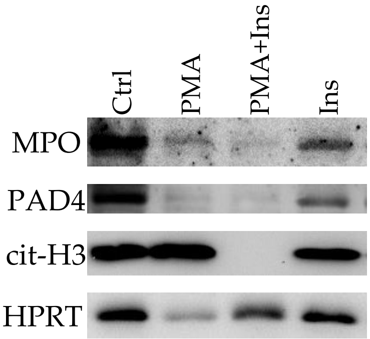

Full Western Blot images for Figure 4F:
Detection of MPO, PAD4 and citrullinated H3 (cit-H3) by Western Blot after 3 h of neutrophil stimulation (addition of 0.5 mM PMSF after 2 h).
Yellow arrows indicate right band, size markers are on the right side of the image.
The exemplary donor shown in the manuscript is highlighted and the corresponding figure shown.
PMA: 100 nM, Ins: Insulin 160 IU/L

Supplementary Figure 3

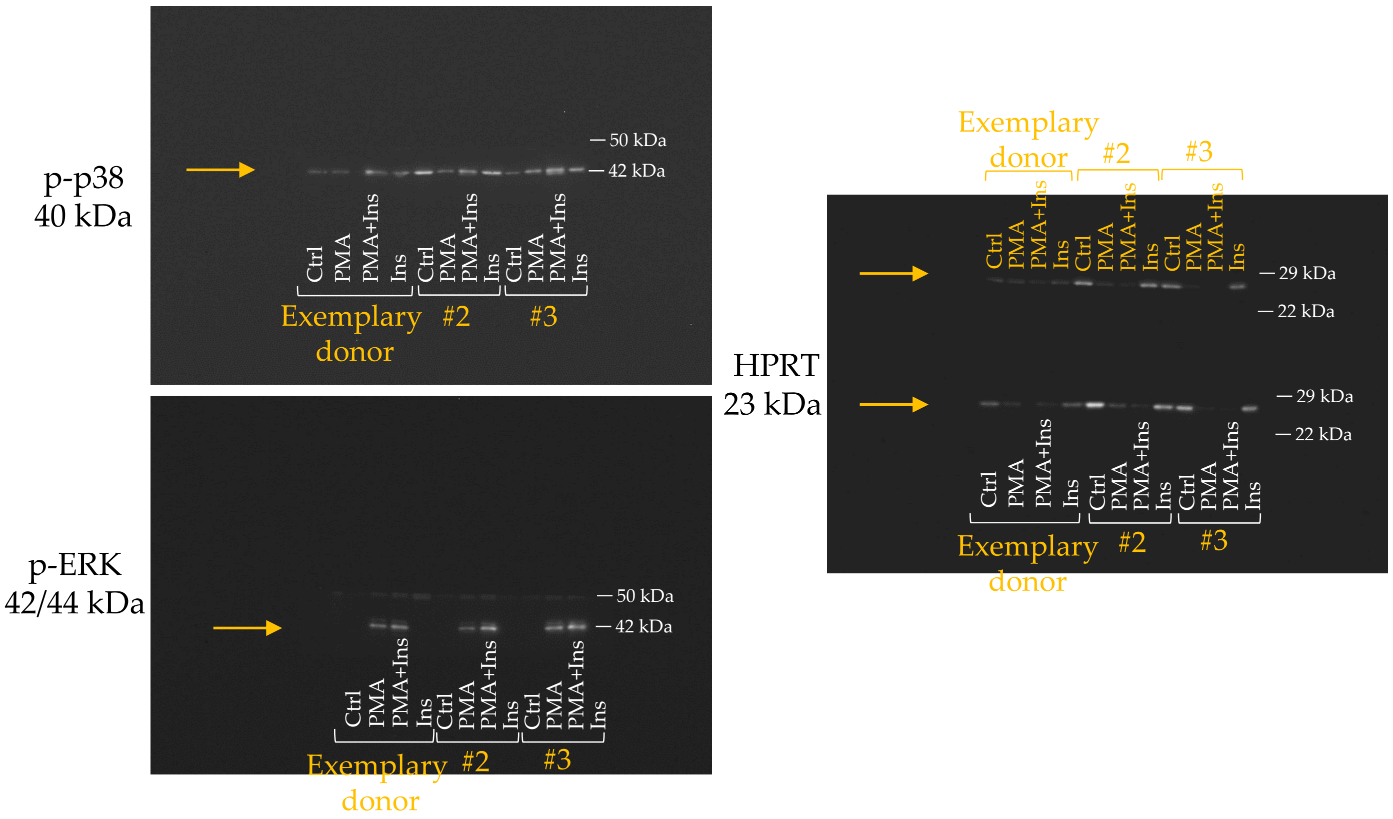

Exemplary donor shown in the supplementary (Supplementary Figure 3)

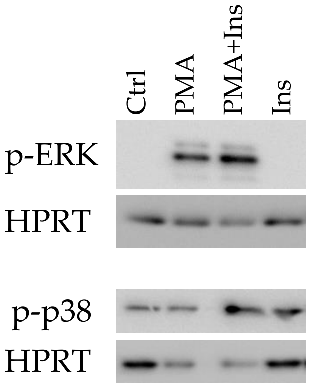

Full Western Blot images for Supplementary Figure 3:
MAPK activation after 1 h stimulation with PMA ± Ins. Western Blot of stimulated neutrophils (1 h) for p-p38 and p-ERK.
Yellow arrows indicate right band, size markers are on the right side of the image.
The exemplary donor shown in the manuscript is highlighted and the corresponding figure shown.
PMA: 100 nM, Ins: Insulin 160 IU/L
