# Supplementary material for: Insulin Can Delay Neutrophil Extracellular Trap Formation In Vitro—Implication for Diabetic Wound Care?
Source: Biology (Basel). 2023 Aug 3;12(8):1082. doi: 10.3390/biology12081082 (PMC10452400; doi:10.3390/biology12081082)
Supplement: Supplementary file 1 [file biology-12-01082-s001.zip › biology-2505194-supplementary/biology-2505194-supplementary-R1/Linnemann2023_Supplementary Figures.pdf]

Insulin can delay neutrophil extracellular trap formation *in vitro* - Implication for diabetic wound care?

Caren Linnemann[1], Filiz Sahin[1], Ningna Li[2], Andreas K. Nussler[1], Stefan Pscherer[1,3], Friedrich Götz[2], Tina Histing[1], Sabrina Ehnert[1,*]

[1] Siegfried Weller Institute for trauma research, BG Unfallklinik Tübingen, Eberhard Karls Universität Tuebingen, Tuebingen, Germany

[2] Microbial Genetics, Interfaculty Institute of Microbiology and Infection Medicine Tübingen, Eberhard Karls Universität Tuebingen, Germany

[3] Department of Internal Medicine III, Sophien- and Hufeland-Hospital, Weimar, Germany;

[*]Correspondence: sabrina.ehnert@gmail.com; Tel.: Tel.: +49 7071 606 1065, fax.: +49 7071 606 1978

*** Corresponding author: Sabrina Ehnert**

Siegfried Weller Institute for trauma research at BG Unfallklinik Tuebingen,

Eberhard Karls Universität Tuebingen

Schnarrenbergstr. 95, 72076 Tuebingen

Tel.: +49 7071 606 1065, fax.: +49 7071 606 1978

E-mail: sabrina.ehnert@gmail.com

Running title: Insulin delays NET formation

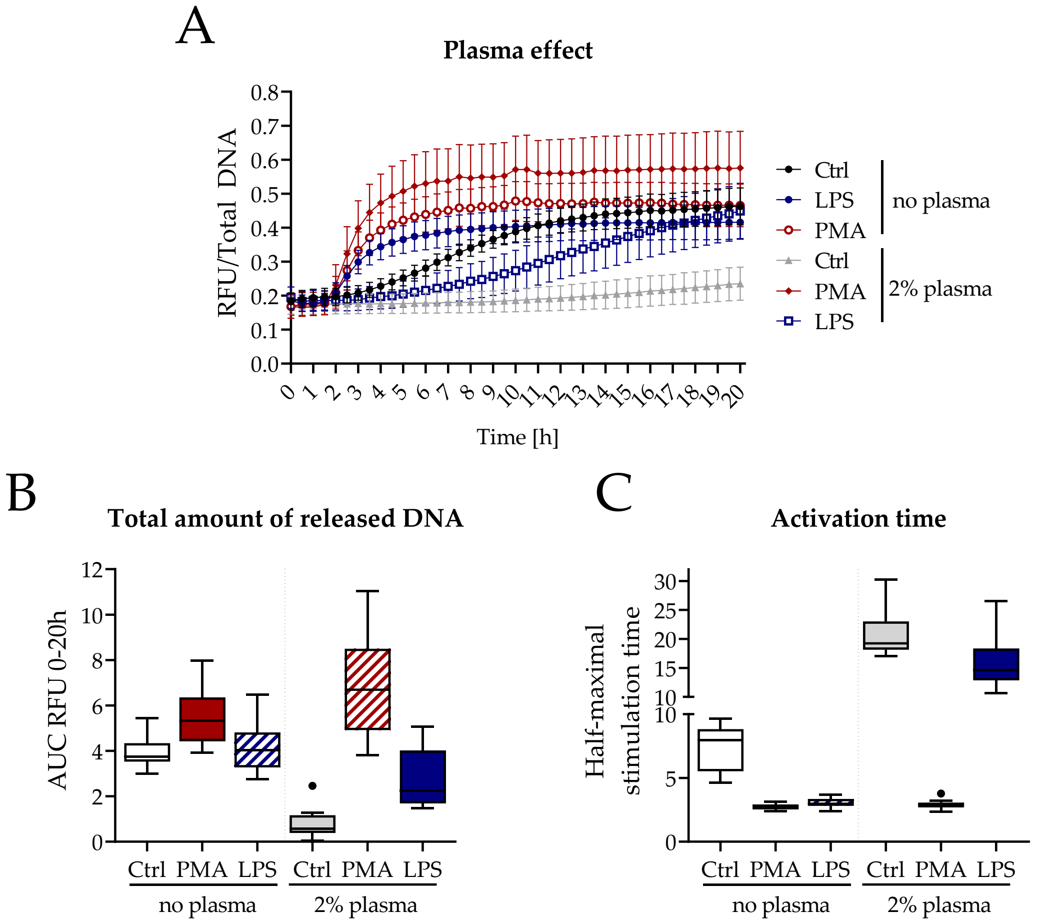

Figure S1: Addition of 2% autologous plasma prevents NET release by LPS **(A)** Time course of Sytox Green Assay measurement 0-20 h, values were normalized to total DNA. **(B)** Total amount of released DNA determined by Sytox Green Assay after stimulation with LPS or PMA ± 2% autologous plasma **(C)** Half-maximal stimulation time determined from Sytox Green Assay. N=4, n=4. LPS 25 µg/mL. PMA: 100 nM.

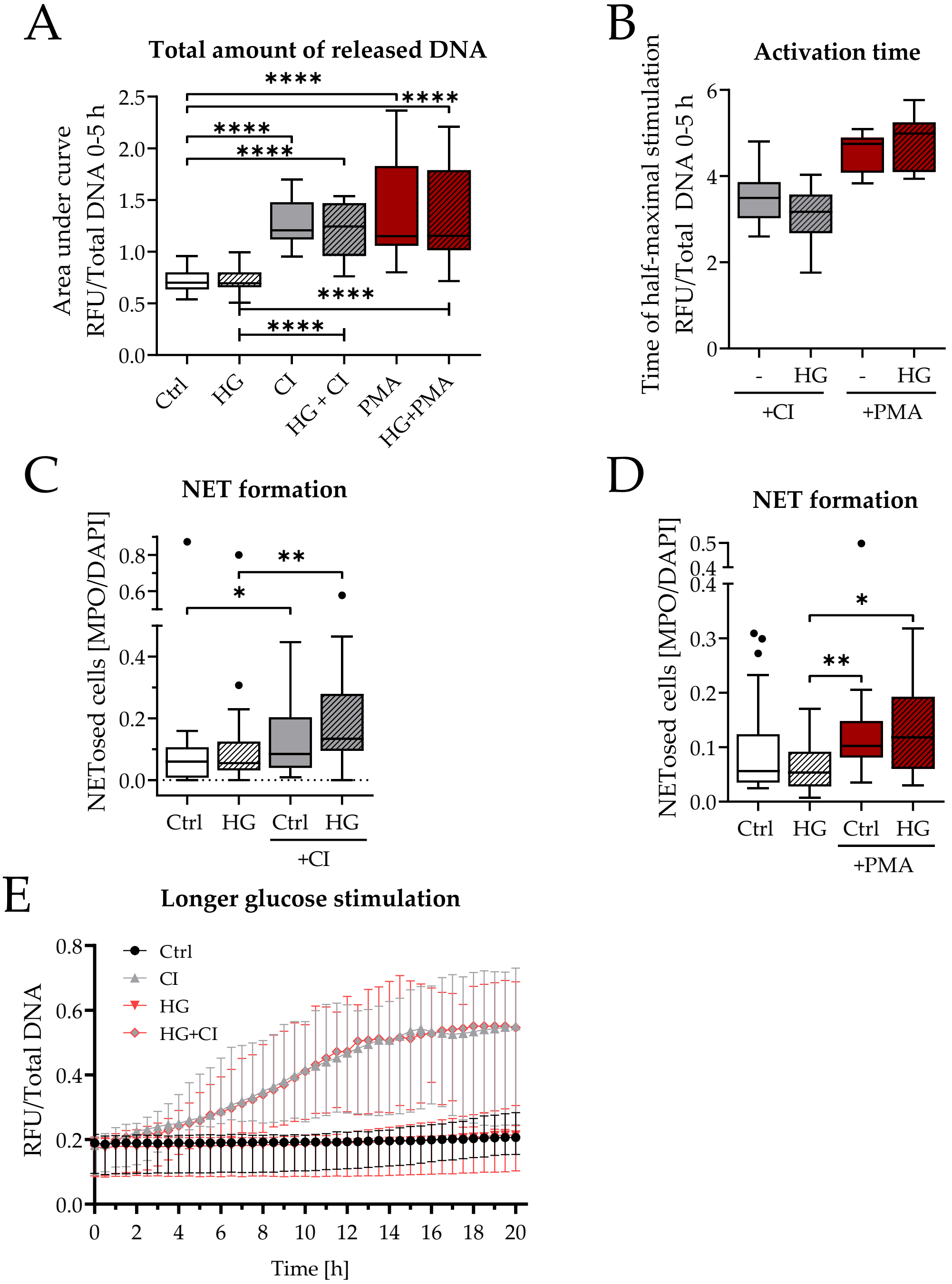

Figure S2: HG does not prime neutrophils for NET release **(A)** Total amount of released DNA determined by Sytox Green Assay after 1 h pre-stimulation with 25 mM glucose and then addition of 100 nM PMA or 4 µM CI **(B)** Half-maximal stimulation time determined from Sytox Green Assay **(C)** NET formation determined by immunofluorescence analysis after 2 h stimulation (1 h pre-stimulation + 1 h CI) **(D)** NET formation determined by immunofluorescence analysis after 3 h stimulation time (1 h pre-stimulation + 2 h PMA) or 3 h (PMA) stimulation time. N=4, n=5. **(E)** Time course of Sytox Green Assay measurement 0-20 h, values were normalized to total DNA. *$p<0.05$, **$p<0.01$,****$p<0.0001$ as determined by Kruskal-Wallis test.

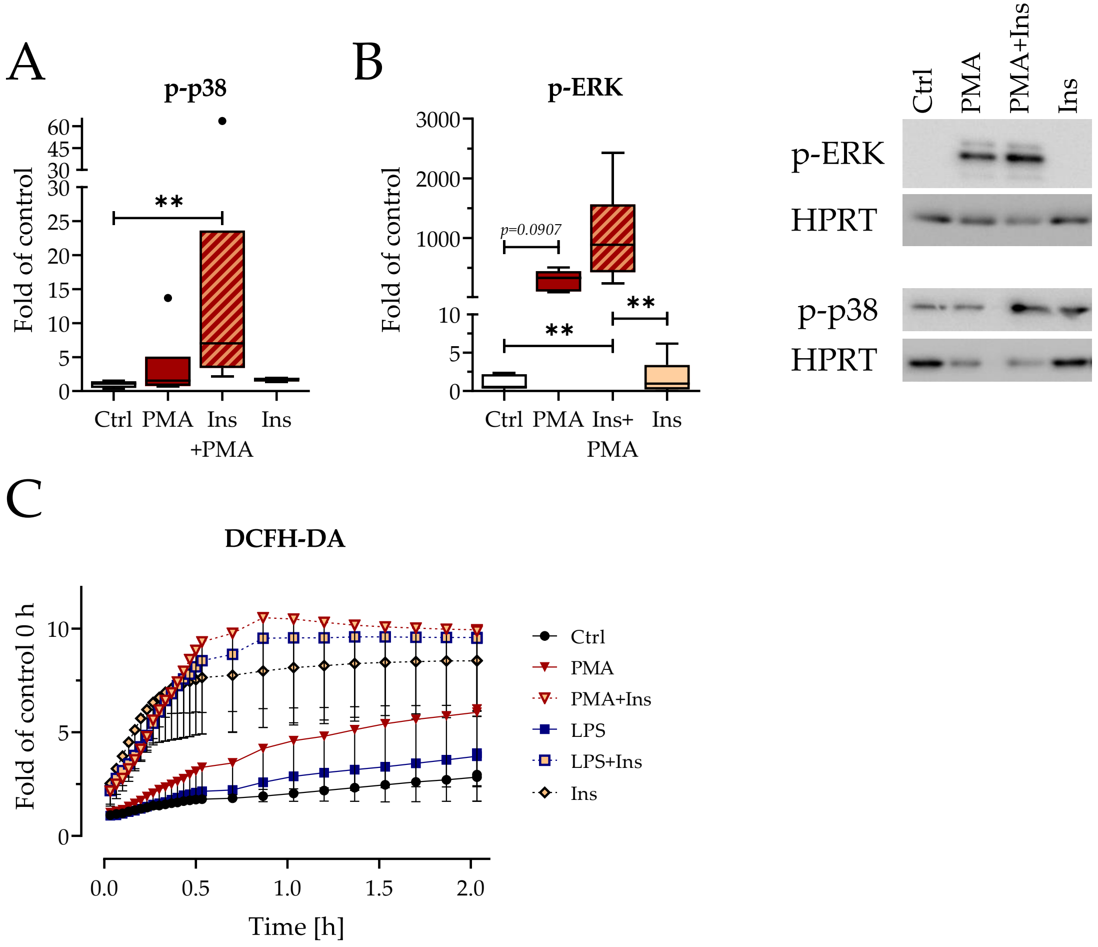

Figure S3: MAPK activation after 1 h stimulation with PMA ± Ins. Western Blot of stimulated neutrophils (1 h). **(A)** p-p38 **(B)** p-ERK. Exemplary blot images are shown on the right. N=4, n=1-2. **(C)** Time course analysis of ROS production in stimulated neutrophils (DCFH-DA staining). N=4, n=3. $^{**}p<0.01$ as determined by Kruskal-Wallis test.
